# The knowledge-behavior gap in nutritional literacy and its associations with chronic disease in older adults: a mediation analysis

**DOI:** 10.3389/fnut.2026.1782729

**Published:** 2026-04-10

**Authors:** Jingyun Zeng, Pinyue Tao, Lichong Lai, Haichen Wu, Shuyu Lu, Dongmei Huang, Pengxin Dong, Huiming Zhou, Zhixin Li, Haowei Liu, Yidan Chai, Ping Huang, Huiqiao Huang

**Affiliations:** 1 Department of Nursing, The Second Affiliated Hospital of GuangXi Medical University, Nanning, China; 2 Department of Anesthesiology, The Second Affiliated Hospital of GuangXi Medical University, Nanning, China; 3 Office of the Party Committee, The Second Affiliated Hospital of GuangXi Medical University, Nanning, China

**Keywords:** chronic disease, knowledge, attitude, and practice model, masking effect, mediation analysis, nutritional literacy, older adult

## Abstract

**Objectives:**

This study employs the Knowledge, Attitude, and Practice (KAP) model to examine the association between nutritional literacy and chronic disease occurrence among older adults in Guangxi, China, aiming to reveal the existence and mechanism of the “knowledge–behavior gap” in the adults' nutritional literacy.

**Methods:**

From May to September 2025, a stratified random sampling method was utilized to conduct a questionnaire survey on nutritional literacy and chronic diseases among 9,249 urban and rural older adults from four cities in Guangxi. Factors influencing nutritional literacy were analyzed through multiple linear regression, and mediation effect testing using bootstrapping with 5,000 resamples.

**Results:**

This study revealed an average nutritional literacy score of 73.60 ± 9.80; besides, totally 4,024 participants (43.82%) suffered from chronic diseases. The total nutritional literacy and “behavior and skills” dimension scores were shown to be significantly higher in healthy older adults than those with chronic diseases (*P* < 0.05); however, their “knowledge and understanding” score was conversely low (*P* < 0.05), which indicated the knowledge–behavior gap. Regression analysis suggested that nutritional literacy knowledge and understanding enhanced both lifestyle/dietary behaviors and basic skills (*P* < 0.05), whereas both lifestyle and dietary behaviors and basic skills were negatively associated with chronic diseases (*P* < 0.05). The mediation effect analysis revealed the following masking effect: Although nutritional literacy knowledge had a positive direct effect on chronic diseases (β = 0.057, SE = 0.007, 95% CI = 0.044~0.071, *P* < 0.001), it exerted a negative total indirect effect on chronic diseases through behaviors and skills (β = −0.047, BootSE = 0.004, 95% Bootstrap CI = −0.056 ~ −0.039).

**Conclusions:**

This study clarifies the existence of a knowledge–behavior gap in the nutritional literacy of older adults in Guangxi and the masking role played by nutritional knowledge and understanding in the relationship between nutritional literacy and chronic disease prevention/management. As a result, chronic disease prevention and management efforts should extend beyond the mere dissemination of theoretical knowledge to encompass intervention strategies centered on behavior promotion and skills training, aiming to realize their potential health benefits.

## 1 Introduction

At present, China is facing the dual public health challenges of population aging and a high incidence of chronic diseases. The age-related decline in metabolic function and the prevalence of multimorbidity make older adults highly susceptible to nutritional imbalances, which in turn exacerbate chronic disease progression and lead to repeated hospitalizations (1). Nutrition literacy, as a vital component of health literacy, is individuals' ability to access, analyze, and understand basic nutritional information and services and utilize them to make sound nutritional decisions to maintain and stimulate their own nutrition and health (2). In December 2025, the Nutrition Literacy Branch of the China Health Promotion and Education Association released a report indicating that merely 11.3% of elderly respondents nationwide possessed adequate nutrition literacy. Moreover, significant imbalances exist across dimensions of nutritional literacy among the elderly: while 41.7% demonstrated cognitive literacy, only 12.7% met the standards for behavioral skills literacy (3). This data suggests a “knowledge-behavior gap” in the nutritional literacy of China's elderly population, demonstrating that despite possessing some nutritional knowledge, they struggle to translate it into practical health behaviors and skills (4). In addition, the phenomenon of “knowing without acting” has been extensively theorized in health behavior research, with explanations ranging from self-regulation deficits to social learning processes (5).

Guangxi, located in western China, is experiencing a progressively deepening degree of aging. There is a lack of large-scale empirical research on the current status of nutritional literacy among older persons and on the association between nutritional literacy and chronic diseases (6). Therefore, based on national initiatives and local needs, this study employs the “Knowledge, Attitudes, and Practices” (KAP) theoretical framework to investigate the associative pathways between nutritional knowledge, behaviors, and skills among Guangxi's elderly population and chronic diseases (7), by employing a cross-sectional survey and chained mediation analysis to examine how knowledge translates into health outcomes via behavioral and skill-based pathways.

## 2 Subjects and methods

### 2.1 Subjects

From May to September 2025, a stratified random sampling method was employed. In the first stage, based on the region's geographical and economic characteristics, four cities including Beihai, Nanning, Laibin, and Guilin were randomly selected (from south to north). In the second stage, based on census data, a 1:1 ratio was employed to select subdistricts (urban areas) or townships (rural areas). The final stage employed convenience or cluster sampling to include eligible elderly individuals willing to participate in the survey from the selected subdistricts and townships. In addition, the allocation of urban and rural sample sizes was determined based on the actual distribution of the elderly population in each region to ensure sample representativeness (8). According to the findings of the “Sixth National Health Service Survey Report, 2021,” the prevalence of chronic diseases among the elderly in China stands at 59.1% (9). By calculating the sample size using the single-population rate sample size formula from epidemiological cross-sectional surveys, and accounting for the design effect and non-response rate, the minimum required sample size is 6,879 (10).

The studies involving humans were approved by The Institutional Review Board of the Second Affiliated Hospital of Guangxi Medical University [2025-KYC(0512)]. The studies were carried out in accordance with the local legislation and institutional requirements. The participants provided their written informed consent to participate in the current study.

The study's inclusion criteria were presented as follows: age ≥ 60 years; conscious, and mentally and intellectually normal; able to understand the questionnaire's content and answer it; and provision of informed consent to participate in this study. The following were the exclusion criteria: (1) substance addiction and obvious memory and intellectual impairment, “substance addiction” refers to self-reported or medically documented dependence on alcohol, illicit drugs, or prescription medications, as assessed by community health workers during the recruitment; (2) obvious hearing impairment, speech communication disorder; as well as (3) failure to complete the questionnaire. In order to guarantee the survey population's representativeness, 10% or more of older adults aged 80 years or older were enrolled.

### 2.2 Survey instrument

The questionnaire included the following four sections: sociodemographic characteristics (age, sex, ethnicity, marital status, education, and residence), lifestyle (smoking and alcohol consumption), the chronic diseases diagnosed by a physician, and nutritional literacy in the older population.

Chronic diseases were defined according to self-reported physician-diagnosed conditions. Participants were asked whether they had ever been diagnosed with any of the following 13 categories: hypertension; dyslipidemia (elevated low-density lipoprotein, triglycerides, or total cholesterol, or low high-density lipoprotein); diabetes or high blood sugar; cancer or malignant tumor (excluding minor skin cancers); chronic lung diseases (e.g., chronic bronchitis, emphysema); liver disease (excluding fatty liver, tumors, and cancer); heart diseases (including heart attack, coronary heart disease, angina, congestive heart failure, or other heart problems); stroke; kidney disease (excluding tumor or cancer); stomach or other digestive diseases (excluding tumor or cancer); memory-related disease; arthritis or rheumatism; and asthma. Based on the number of reported conditions, participants were categorized as “healthy” (none), “single chronic disease,” or “multiple chronic diseases” (≥2) (11).

Nutrition literacy assessment employed the Chinese Elderly Nutrition Literacy Questionnaire developed by Professor Zhang Zhaofeng of Peking University's School of Public Health in 2022. The questionnaire demonstrated robust validity and reliability overall (S-CVI = 0.95, I-CVI ≥ 0.83, Kappa > 0.74, Cronbach's α = 0.931). This is a nutrition literacy survey questionnaire which is especially developed for the elderly population in China. The questionnaire comprises totally 20 items across three dimensions: Knowledge and Understanding (35 points), Lifestyle and Dietary Behavior (45 points), and Basic Skills (20 points), with a total score of 100 points. Among the elderly population, higher scores indicate greater mastery of nutrition and health knowledge (12, 13). Similar approaches to food literacy measurement have been validated across diverse cultural settings, exhibiting the robustness of such multi-dimensional constructs (14, 15).

### 2.3 Survey methods

A face-to-face survey was conducted by social workers at all levels of the community and townships and by medical and nursing staff who had been uniformly trained and were familiar with the local dialect. The survey used a uniform instruction. After obtaining participants' consent, the survey was conducted such that questions were asked by the surveyor and answered by the older participants. Finally, all the involved questionnaires were collected on the spot.

### 2.4 Statistical methods

Data were analyzed through SPSS version 27.0 (IBM Corp., Armonk, NY, USA). Measurement data are indicated as mean ± standard deviation or median (interquartile range), and categorical data as frequencies and percentages. Differences in nutritional literacy among older adults were assessed using independent samples t-tests and one-way ANOVA. Multiple linear regression was employed to identify factors associated with nutritional literacy. Mediation analysis was performed through Hayes' PROCESS macro (v4.2, Model 6), which estimates chained mediation effects with multiple mediators and provides bias-corrected bootstrap confidence intervals. Besides, this model was selected to test the hypothesized sequential pathway (knowledge → lifestyle/dietary behaviors → basic skills → chronic disease). All models controlled for potential confounding by covariates including age, gender, educational level, marital status, living alone status, nutritional supplement use, as well as health status. Prior to the main analysis, multicollinearity was assessed using the variance inflation factor (VIF); all VIF values were below 3, suggesting no serious concerns. Heteroscedasticity was examined using the Breusch–Pagan test, and the result was not significant (*p* > 0.05), satisfying the assumption of homoscedasticity. Bootstrap resampling with 5,000 iterations was employed to generate 95% confidence intervals for indirect effects; an interval excluding zero represented statistical significance at α = 0.05.

**Figure 1:**
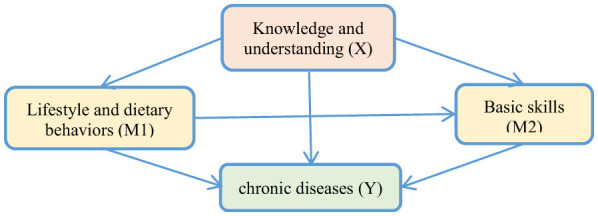
A chained mediation model for the effect of older adults' nutritional literacy on chronic disease.

Figure 1 illustrates the hypothesized chained mediation model. In this model, the “knowledge and understanding” dimension of nutritional literacy **(X)** is proposed to impact chronic disease **(Y)** through two sequential mediators: “lifestyle and dietary behaviors” (M1) and “basic skills” (M2). The model specifies three indirect pathways: (1) X → M1 → Y, indicating the mediating effect of lifestyle and dietary behaviors; (2) X → M2 → Y, representing the mediating effect of basic skills; and (3) X → M1 → M2 → Y, the chained mediation pathway, where knowledge influences behaviors, which in turn enhance skills, ultimately influencing chronic disease. The direct path (X → Y) is also estimated, representing the remaining effect of knowledge on chronic disease after accounting for the mediators.

## 3 Results

### 3.1 General information

In this study, totally 9,702 questionnaires were distributed and 9,249 valid questionnaires were returned (95.3%). Furthermore, altogether 9,249 people (aged 60–107 years, average age = 71.696 ± 0.18), comprising 4,357 men (47.1%) and 4,892 women (52.9%), participated in this study. The older population's average nutritional literacy score, prevalence of disease rate, and prevalence of comorbidity rate were shown to be 73.609 ± 0.80, 43.86, and 21.2%, respectively.

### 3.2 Comparison of nutritional literacy among older adults

A comparison of nutritional literacy among older adults with different sociodemographic backgrounds, including different age, gender, education, marital status, health status, residence status, and nutritional supplement intake, revealed statistically significant differences (*P* < 0.05), as presented in Table 1.

**Table 1:** Comparison of total nutritional literacy scores of older adults from different sociodemographic backgrounds.

Demographic variable	Number of persons	Total nutritional literacy score for older adults	*t/F*	*p*
Gender			2.003	0.045
Men	4,357	73.829 ± 0.70		
Women	4,892	73.409 ± 0.88		
Age group (years)			2.691	0.007
60–79 (Year)	7,394	74.019 ± 0.89		
80 and above	1,015	73.071 ± 0.55		
Educational level			111.564	<0.001
Uneducated	1,118	71.099 ± 0.20		
Primary and below	5,034	72.709 ± 0.21		
Middle school	2,181	75.529 ± 0.66		
High school	699	77.661 ± 0.69		
University and above	217	80.609 ± 0.80		
Marital status			4.276	0.002
Unmarried	95	71.958 ± 0.95		
Married (with partner)	7,436	73.669 ± 0.97		
Divorced	109	72.681 ± 1.13		
Separated	27	66.788 ± 0.55		
Widowed	1,582	73.588 ± 0.90		
Health status			8.952	<0.001
Health	5,196	73.969 ± 0.20		
Single chronic disease	2,066	72.591 ± 0.51		
Multiple chronic diseases	1,958	73.661 ± 0.45		
Cancer	29	74.961 ± 0.70		
Living alone			2.691	0.007
Yes	1,855	73.071 ± 0.55		
No	7,394	74.019 ± 0.89		
Smoking			0.463	0.644
Yes	1,623	73.709 ± 0.47		
No	7,626	73.589 ± 0.87		
Alcohol consumption			0.336	0.737
Yes	1,642	73.679 ± 0.44		
No	7,607	73.589 ± 0.88		
Nutritional supplement intake			12.178	<0.001
Yes	3,163	75.311 ± 0.16		
No	6,086	72.719 ± 0.49		

The older population's total nutrient literacy score was the dependent variable, with age, education, gender, living alone status, and nutritional supplement use being used as independent variables. Moreover, marital status (with married as the reference group) and health status (with healthy as the reference group) were analyzed through multiple linear regression, which was of statistical significance (*F* = 40.794, *P* < 0.001) and explained 5.5% of the variance in the total nutritional literacy score. Results suggested that education was the strongest positive predictor of nutritional literacy (β = 2.170, *P* < 0.001), and consuming nutritional supplements was independently and negatively associated with nutritional literacy (β = −2.350, *P* < 0.001). Regarding marital status, separated older adults had significantly lower nutritional literacy scores (β = −5.893, *P* < 0.05) and widowed elderly had obviously higher literacy scores (β = 0.863, *P* < 0.05) compared with married elderly. In terms of disease health status, the total nutrient literacy scores were lower older adults having a single chronic disease (β = −1.313, *P* < 0.001) and in those with multiple chronic diseases (β = −0.575, *P* < 0.05) compared with healthy elderly. Besides, the effects of age, gender, living alone status, and other marital and disease statuses did not exhibit statistical significance (Table 2).

**Table 2:** Results of the multiple linear regression analysis of factors influencing older adults' nutritional literacy.

Independent variable	Reference group	β	Standard error	*β^′^*	*t*	*p*
Constant		72.620	1.722	—	42.183	0.000
Age (year)		−0.005	0.018	−0.003	−0.264	0.792
Academic qualification		2.170	0.130	0.187	16.661	<0.001
Gender	Male (cf. female)	0.061	0.219	0.003	0.278	0.781
Living alone	Yes (cf. No)	0.327	0.40	0.010	0.804	0.421
Nutritional supplement use	Yes (cf. No)	−2.350	0.226	−0.112	−10.388	<0.001
Marital status	(Reference: married)					
Unmarried		−0.531	1.082	−0.005	−0.491	0.624
Divorced		−1.608	0.965	−0.018	−1.666	0.096
Separated		−5.893	2.605	−0.024	−2.263	0.024
Widowed		0.863	0.321	0.032	2.684	0.007
Health status	(Reference: health)					
Single chronic disease		−1.313	0.268	−0.055	−4.908	<0.001
Multiple chronic diseases		−0.575	0.274	−0.024	−2.100	0.036
Cancer		0.263	1.948	0.001	0.135	0.893

R^2^ = 0.055, adjusted R^2^ = 0.054, F = 40.794, p <0.001

### 3.3 Comparison of total nutritional literacy and variable scores between healthy and chronically ill elderly

In the knowledge and understanding dimension, the older adults with multiple chronic diseases (26.024 ± 0.09) obtained the highest scores. Besides, in the lifestyle and dietary behavior and basic skills dimensions, the highest scores were obtained by healthy elderly (33.374 ± 0.83 and 14.962 ± 0.39, respectively). which were significantly higher than those of older adults with either single or multiple chronic conditions (*P* < 0.05). Finally, regarding total nutritional literacy, healthy elderly had the highest score (73.969 ± 0.20) and the older adults with a single chronic disease had the lowest (72.591 ± 0.51; *P* < 0.05). Results revealed a typical knowledge–behavior disconnection phenomenon, as listed in Table 3.

**Table 3:** Comparison of nutritional literacy variable scores among healthy older adults and those with chronic diseases (x ± s).

Variables	Healthy elderly (5,196)	Single chronic disease (2,066)	Multiple chronic diseases (1,958)	*F*	*P*	*Post-hoc* comparisons (*P* < 0.05)
Knowledge and understanding	25.634 ± 0.01	25.763 ± 0.94	26.024 ± 0.09	4.852	0.001	Healthy group <Multiple chronic diseases group. Single chronic disease group <Multiple chronic diseases group.
Lifestyle and dietary behaviors	33.374 ± 0.83	32.155 ± 0.76	32.985 ± 0.36	21.714	<0.001	Healthy group > Single chronic disease group. Healthy group > Multiple chronic diseases group. Multiple chronic diseases group > Single chronic disease group.
Basic skills	14.962 ± 0.39	14.682 ± 0.43	14.662 ± 0.63	10.230	<0.001	Healthy group > Single chronic disease group. Health group > Multiple chronic diseases group.
Total nutrient literacy score	73.969 ± 0.20	72.591 ± 0.51	73.661 ± 0.45	8.952	<0.001	Healthy group > Single chronic disease group. Multiple chronic diseases group > Single chronic disease group.

### 3.4 Regression and mediation analyses

Prior to the main mediation analysis, a chi-square test was carried out to examine the association between nutritional supplement use and chronic disease status. The results revealed a significant association (χ^2^ = 43.88, *P* < 0.05), with supplement use being more prevalent among older adults with chronic diseases 37.9% (1,536/4,053) relative to their healthy counterparts 31.3% (1,627/5,196). Moreover, this suggests that individuals with poorer health are more likely to turn to nutritional supplements, consistent with the “health need effect” observed in subsequent analyses (16).

After controlling for demographic and other confounders, regression analyses indicated that knowledge and understanding of nutritional literacy positively predicted both lifestyle and dietary behaviors (β = 0.753, 95%CI = 0.731~0.775, *P* < 0.001) and basic skills (β = 0.115, 95%CI = 0.102~0.128, *P* < 0.001). In turn, both lifestyle and dietary behaviors and basic skills were negatively correlated with chronic diseases (behavior: β = −0.052, 95%CI = −0.063 ~ −0.041, *P* < 0.001; skills: β = −0.031, 95% CI = −0.052 ~ −0.011, *P* < 0.05).

Besides, mediation analysis revealed a more complex pattern: After controlling for behaviors and skills, knowledge of nutritional literacy exhibited a positive direct association with chronic diseases (β = 0.057, SE = 0.007, 95% CI = 0.044~0.071, *P* < 0.001). Nevertheless, knowledge exerted a significant negative indirect effect on chronic diseases through behaviors and skills (β = −0.047, BootSE = 0.004, 95% Bootstrap CI = −0.056 ~ −0.039). The opposite directions of the direct and indirect pathways conform to a statistical “masking effect” (17), as detailed in Table 4.

**Table 4:** Mediation analysis of nutritional literacy variables on chronic disease after adjusting for covariates (*N* = 9,249).

Paths/variables	Model	*β/Log-odds*	Standard error	*t/Z*	*P*	95% confidence interval
1. Predicting lifestyle and dietary behaviors (M1)						
Constants	Linear regression	12.967	0.756	*t =* 17.15	<0.001	11.484 ~14.449
Knowledge and understanding (X)		0.753	0.011	*t =* 67.88	<0.001	0.731 ~ 0.775
2. Forecasting basic skills (M2)						
Constants	Linear regression	6.398	0.375	*t =* 17.07	<0.001	5.664 ~7.133
Knowledge and understanding		0.115	0.007	*t =* 17.38	<0.001	0.102 ~ 0.128
Lifestyle and dietary behaviors		0.198	0.005	*t =* 39.06	<0.001	0.188 ~ 0.208
3. Predicting chronic diseases (Y)						
Constants	Logistic regression	–2.356	0.386	*Z = –*6.10	<0.001	–3.113 ~–1.599
Knowledge and understanding		0.057	0.007	*Z =* 8.17	<0.001	0.044 ~ 0.071
Lifestyle and dietary behaviors		–0.052	0.006	*Z = –*9.21	<0.001	–0.063 ~–0.041
Basic skills		–0.031	0.011	*Z = –*2.96	0.003	–0.052 ~–0.011
4. Direct and indirect effects						
Direct effect	X → Y	0.057	0.007		<0.001	0.044 ~ 0.071
Total indirect effects		−0.047	0.004			–0.056 ~–0.039
Indirect effect 1	X → M1 → Y	–0.039	0.004			–0.048 ~–0.031
Indirect effect 2	X → M2 → Y	–0.004	0.001			–0.006 ~–0.001
Indirect effect 3	X → M1 → M2 → Y	–0.005	0.002			–0.008 ~–0.002

## 4 Discussion

This study's cross-sectional design suggests a bidirectional effect between knowledge and health status, including both the theoretical pathway of knowledge for behavioral change and the reverse process of active knowledge acquisition for health management after illness. As a result, rather than establishing a unidirectional causal relationship, the mediation analysis performed in the current work revealed the complex network of associations between nutritional literacy dimensions and chronic disease to demonstrate the “knowledge-behavior gap” phenomenon and quantify the indirect effects of knowledge on health outcomes through behavioral and skill assessments (18). The study indicated that nutritional literacy in the older population was influenced by multiple factors. Particularly, multiple linear regression analyses suggested that educational attainment, nutritional supplement use, marital separation and widowhood status, and disease status independently influenced nutritional literacy among older adults.

### 4.1 Influence of sociodemographic factors on nutritional literacy in old age

Consistent with Liu et al. (19), and Greenberg et al. (20), educational attainment emerged as the strongest positive predictor of nutritional literacy, underscoring its role as a key driver of nutrition knowledge in older adults. Considering that this population usually holds established knowledge structures, intergenerational learning as suggested by So et al. (21) may provide an effective pathway for literacy enhancement through integrating nutrition education into school curricula and fostering family-based information exchange.

Marital status also exerted a significant role. Separated older adults exhibited lower nutritional literacy, probably due to disruptions in social support systems that facilitate health information acquisition (22). By contrast, widowed individuals scored higher than their married counterparts, possibly because the necessity of independent living after loss heightens attention to personal health management. This form of adaptive learning merits further exploration.

In terms of health status, although older adults with chronic diseases, particularly those with multimorbidity, scored higher on the knowledge dimension than healthy peers (Table 3), their performance on behavioral and skills-based dimensions was remarkably lower (*P* < 0.001). This obvious knowledge-behavior gap suggests that while chronic disease may serve as a catalyst for information-seeking (23, 24), translating that knowledge into daily practice remains a challenge. As demonstrated by Rimal (25), the translation of health knowledge into actual behavior is critically mediated by individuals' self-efficacy, which is the perceived ability to exert personal control over health practices. Without corresponding confidence and practical skills, even substantial knowledge may fail to produce behavioral change. Indeed, nutrition interventions in China continue to underscore knowledge dissemination over skill-building (24). Bridging this gap likely requires structured external support, as observed in initiatives like the Healthy Families program (22).

After controlling for factors including educational attainment, supplement use was negatively associated with nutritional literacy. This pattern is consistent with the health demand effect reported by Khawagi et al. (26), wherein older adults in poorer health turn to supplements despite limited nutritional knowledge. By contrast, those with higher literacy tended to adhere to the traditional belief that dietary therapy is superior to medication, favoring balanced diets over supplements. This preference for dietary diversity aligns with the Lingnan dietary pattern prevalent in neighboring Guangdong (27), a region sharing culinary similarities with Guangxi. Furthermore, this cultural inclination is reinforced by systematic reviews, emphasizing that age-appropriate, nutrient-dense dietary patterns are foundational to preventing malnutrition and chronic disease in later life (28).

### 4.2 Integrated explanation of the knowledge–activity disconnect mechanism

#### 4.2.1 Critical reading of positive associations: the dominant role of reverse causation

This study observed a key finding in the mediation model: after controlling for skills and behaviors, nutritional knowledge was positively associated with chronic disease. Owing to the cross-sectional design, this likely reflects reverse causality (29), that is, older adults with chronic disease become more proactive in seeking nutritional knowledge due to heightened health management needs.

This interpretation is theoretically grounded in the Health Belief Model (HBM) and Theory of Planned Behavior (TPB). In accordance with the HBM, heightened perceived disease threat following diagnosis motivates health-related actions, including information-seeking (30).The TPB further suggests that diagnosis may shift attitudes, strengthen normative beliefs about healthy eating, and enhance perceived behavioral control, collectively driving engagement with nutritional information (31). Therefore, the positive association observed likely reflects a disease state → knowledge learning pathway rather than knowledge causally leading to disease. Although alternative explanations including optimism bias (32), information anxiety (33), or echo chamber effects (34) have been proposed, our data did not include relevant psychological measures to test these mechanisms, warranting future longitudinal research with richer variable sets.

#### 4.2.2 Validation of the core protection pathway: transformation of knowledge into behavior and skills

In contrast to the positive direct association discussed above, knowledge exerted a significant negative indirect effect on chronic disease through lifestyle and dietary behaviors and basic skills. The strongest effect emerged in the knowledge → behavior → chronic disease pathway, indicating that translating nutritional knowledge into daily practice is the most direct route to reducing disease risk. This finding underscores the importance of behavioral and skill-based mediators in the knowledge-health relationship, consistent with research demonstrating that perceived benefits and self-efficacy are key predictors of engagement in preventive health behaviors (35). Unlike the disease-driven knowledge acquisition described in 4.2.1, this pathway represents the protective function of knowledge when successfully translated into action—a distinction central to understanding the masking effect observed in this study.

The knowledge → skills → chronic disease pathway further highlights the significance of converting abstract knowledge into practical skills including food label reading and meal planning—a process facilitated by social interaction and knowledge transfer, as noted in Zhou et al. (36) social cognitive theory–based study. Finally, the sequential knowledge → behavior → skill → disease pathway illustrates a dynamic, mutually reinforcing cycle between behavior and skill development. This is consistent with Zhuge et al.'s (37) extended health behavior model, which conceptualizes health behavior formation as a cognitively driven, practice-based process that ultimately internalizes as personal capability.

#### 4.2.3 Practical implications of the masking effect for nutritional interventions

The masking effect identified in the present study holds significant practical implications for enhancing nutrition among older adults (38). When nutritional knowledge fails to translate into dietary behaviors and practical skills, its potential health benefits are diminished. This finding suggests that traditional one-way knowledge dissemination models, such as lectures and pamphlet distribution, are insufficient for chronic disease prevention. These approaches address the knowing aspect but fail to bridge the gap between knowledge and action. As a result, nutrition interventions for the elderly must shift toward behavior-oriented comprehensive models highlighting the following strategies.

(1) Contextualized behavioral interventions. It is essential to design concise and actionable dietary guidelines tailored to Guangxi seniors' eating habits, including pairing local ingredients, portion control, and lowering high-salt or high-oil cooking methods. On-site demonstrations and hands-on training can help translate nutritional knowledge into daily practice.
(2) Skill training tailored to practical needs. It is vital to focus on core competencies essential for independent living, including interpreting food labels, identifying nutritional components, creating simplified meal plans for chronic disease patients, and selecting appropriate supplements (39). Small-scale interactive skill workshops can enhance practical abilities, reinforcing the mediating role of skills in the knowledge-to-health pathway.
(3) Long-term behavioral monitoring with social support. It is necessary to establish a multi-tiered support system involving community healthcare providers, family members, and peer volunteers to conduct regular health behavior follow-ups, dietary tracking, and mutual monitoring (40). Utilizing senior dining halls can also address practical challenges including living alone or cooking difficulties, thereby sustaining healthy eating habits over time.
(4) Precision interventions tailored to demographic characteristics. It is important to implement stratified interventions addressing variations in nutritional literacy based on educational background, marital status, and disease status like employing intuitive graphic methods for those with lower education, strengthening one-on-one guidance for socially isolated individuals, and focusing on behavioral skills training for chronic disease patients with strong knowledge but weak execution.

### 4.3 Limitations

However, the following limitations should be acknowledged. At first, the cross-sectional design precludes causal inference. Reverse causality remains possible, highlighting the need for longitudinal studies to confirm causal pathways. Secondly, both chronic disease status and nutritional literacy were self-reported, which may introduce recall or social desirability bias. Future research should incorporate objective measures like clinical examinations or direct observation. Thirdly, the sample was restricted to four cities in Guangxi, limiting the generalizability of findings to other regions or populations. Multicenter studies across broader geographic areas are needed to validate these results. In addition, unmeasured factors—including psychological variables, social support, and environmental determinants—may influence nutritional literacy levels and warrant further investigation. Fourthly, the prevalence of multimorbidity in our study (21.2%) was lower than that reported in many epidemiological studies from high-income countries. This discrepancy may be attributed to: (1) the reliance on self-reported physician-diagnosed conditions, which may underestimate true prevalence compared to clinical assessments or medical records; (2) the inclusion of relatively healthy community-dwelling older adults, excluding those in medical care facilities or patients suffering from severe disabilities; and (3) potential regional variations in healthcare access and diagnostic practices in western China. Moreover, further studies should incorporate objective clinical assessments and medical record reviews to capture a more comprehensive picture of chronic disease burden.

## 5 Conclusions

To conclude, this study reveals a significant knowledge-behavior gap in nutritional literacy among older adults in Guangxi, characterized by a masking effect where knowledge positively correlates with chronic disease directly but also exerts protective effects indirectly through behaviors and skills. Educational attainment emerged as the strongest positive predictor of nutritional literacy, while supplement use and separation status were negatively associated, and widowhood exhibited higher literacy levels. The validated protective pathway of knowledge transforming into behaviors and skills underscores that nutritional knowledge must be translated into action to benefit health outcomes. In addition, future interventions should prioritize skill development and behavior change over mere knowledge dissemination, with attention to demographic variations, aiming to effectively bridge this gap and improve geriatric health.

## Data Availability

The raw data supporting the conclusions of this article will be made available by the authors, without undue reservation.
